# Brian 2: neural simulations on a variety of computational hardware

**DOI:** 10.1186/1471-2202-15-S1-P199

**Published:** 2014-07-21

**Authors:** Dan FM Goodman, Marcel Stimberg, Pierre Yger, Romain Brette

**Affiliations:** 1 Department of Otology and Laryngology, Harvard Medical School, Boston, MA, 02114, USA; 2 Eaton-Peabody Laboratories, Massachusetts Eye and Ear Infirmary, Boston, MA, 02114, USA; 3 Institut d’Etudes de la Cognition, Ecole Normale Supérieure, Paris, France; 4 Sorbonne Universités, UPMC Univ. Paris 06, UMR_S 968, Institut de la Vision, Paris, F-75012, France; 5 INSERM, U968, Paris, F-75012, France; 6 CNRS, UMR_7210, Paris, F-75012, France

## 

Brian 2 is a fundamental rewrite of the Brian [1,2] simulator for spiking neural networks. It is written in the Python programming language and focuses on simplicity and extensibility: neuronal and synaptic models can be described using mathematical formulae and with the use of physical units [3]. The same formalism can also be used to specify connectivity patterns (e.g. spatial connectivity), using mathematical expressions to define connections, probabilities of connections, number of synapses between neurons, and synaptic delays.

Brian 2 offers two modes of operation: a “runtime mode”, where executable code is generated from the model descriptions on the fly and executed from Python and a “standalone mode”, where a set of source code files is generated that can then be compiled and executed with no dependency on Brian or Python. The runtime mode is ideal for rapid prototyping and interactive exploration, e.g. from a Python console. The standalone mode on the other hand is designed for maximum of performance and for simulating models on a variety of hardware and platforms.

We show a number of example applications for the standalone mode, generating code for a wide range of devices:

• C++ code that is completely independent of Brian, Python or Python libraries. Optionally, this code can be parallelized over multiple CPU cores using the OpenMP libraries.

• Java/Renderscript for Android-based devices, enabling Brian to run neural models on commodity hardware (e.g. phones) for robotic applications [5].

• The GPU enhanced Neuronal Networks (GeNN) framework [6,7], a neural simulator using GPUs to accelerate neural simulations.

• The same approach would also allow the generation of code targeted at neuromorphic computing architectures such as the SpiNNaker platform [8] for which we have started preliminary work.

Brian is made available under a free software license and all development takes place in public code repositories [9].
